# Study on Properties of Glass-Fiber-Fabric-Reinforced Microwave-Absorbing Composites

**DOI:** 10.3390/ma17071453

**Published:** 2024-03-22

**Authors:** Zhuohui Zhou, Yang Liu, Xi Chen, Zhiyong Wang, Yan Zhao

**Affiliations:** 1 AECC Beijing Institute of Aeronautical Materials, Beijing 100095, China; aeccliuyang@126.com (Y.L.); chenxi@126.com (X.C.); zywang91@163.com (Z.W.); 2 School of Materials Science and Engineering, Beihang University, Beijing 100191, China; jennyzhaoyan@buaa.edu.cn

**Keywords:** microwave absorption, glass fiber reinforcement, composite material

## Abstract

In this paper, the glass-fiber-fabric-reinforced resin-based absorbing composites were prepared, and their microwave-absorbing properties were studied via simulation and experiment. The simulation results show that the absorption bandwidth of the absorbing material can cover the X\C\S band, respectively, at different thicknesses. The minimum reflection loss (RL) of the composite with a thickness of 2.2 mm is −27.4 dB at 5.95 GHz. However, the experiment results are quite different from those of the simulation. The metallographic results indicate that it is the change of the mass fraction of the absorbents in the composites after curing that causes the difference. According to the metallographic results, three shape approximation methods were proposed to calculate the real mass ratio of the absorbents in the composites, namely, parallelogram approximation, bows approximation, and elliptical approximation. Meanwhile, the structural parameter $K_{f}$ was introduced to optimize the calculation results. The electromagnetic parameters of the material based on the calculation results were measured, and the results show that the simulation results obtained via bow approximation have a better coincidence to the experiment results, and the mass ratio of the absorbent raises by around 9.95%, which lays a foundation for the subsequent design of microwave-absorbing composites.

## 1. Introduction

The increasing reliance on wireless communication and the proliferation of electronic devices means that people are constantly surrounded by electromagnetic waves. This constant exposure raises concerns about the long-term effects on human health and calls for further research into mitigating these potential risks. In addition to impacting human health, electromagnetic wave pollution can also interfere with other technologies such as medical equipment and aviation systems. As our society becomes increasingly dependent on wireless communication and electronic devices, it is crucial to address the issue of electromagnetic wave pollution in order to ensure a safe and healthy environment for everyone [1,2,3]. Microwave-absorbing material (MAM) is capable of reducing microwave transmission by converting electromagnetic waves into thermal energy through electromagnetic loss [4,5,6]. After decades of development, MAM has evolved into various forms including coatings, foams, honeycombs, and composite materials [7,8,9]. Microwave-absorbing composite materials (MACM) are a type of advanced composites that exhibit strong designability [10,11], exceptional microwave-absorbing performance [12,13], and remarkable corrosion resistance. MACM not only exhibit excellent absorbing performance but also possess a certain mechanical strength, enabling the simultaneous realization of microwave-absorbing properties and load-bearing capabilities. These materials have emerged as the primary focus for the development of microwave-absorbing materials due to their outstanding characteristics [14,15,16,17]. However, in contrast to the prevalent use of continuous carbon fiber as reinforcement in advanced composite materials, the MACM primarily employ woven fibers with transmitting properties, such as glass fiber (GF) and aramid fiber [18,19,20]. By adding microwave-absorbent materials to the resin of the composite, researchers have successfully determined the MACM that can realize microwave absorption properties in different frequency bands. Those absorbents can mainly be divided into three categories, which are dielectric absorbent [21,22,23], magnetic absorbent [24,25,26], and hybrid absorbent [27,28]. Zheng, X. L. et al. [29] prepared FeCuNbSiB powder/S-glass-fiber-reinforced epoxy composite panels via the mold pressing method. The composite with an FeCuNbSiB/epoxy mass ratio of 2.5:1.0 has excellent microwave absorption properties, with a minimum reflection loss value −30.5 dB at 10.93 GHz for a thickness of 2 mm. Chen, W. et al. [30] investigated the microwave absorption and mechanical properties of short-cut carbon fiber/glass fiber hybrid veil-reinforced epoxy composites. The optimum microwave absorption properties can be obtained when the content of CFs in the hybrid veil is 6 wt% and the thickness of the composites is 2 mm; the minimum reflection coefficient of −31.8 dB and the effective absorption bandwidth is 2.1 GHz. Many researchers mainly focus on the direction of improving the absorption and mechanical properties of composite materials, such as adding different kinds of absorbers, including carbon materials and magnetic materials. Chen, W. et al. [31] applied the liquid molding process to manufacture the glass fiber epoxy composites containing multi-walled carbon nanotubes (MWCNTs) and Fe_3_O_4_ NPs. The optimized microwave-absorbing properties of the composites with minimum RL of −45.7 dB, and full X-band effective absorption can be achieved when the total thickness of the matching layer and absorbing layer is 1.8 mm. Liu, Y. et al. [32] synthesized one type of GF hybrid with carbon nanotubes (CNTs) and nickel (Ni). By changing the reaction conditions, the morphology of the GF hybrids, including CNT’s length, CNT’s growth density, and Ni’s thickness, could be adjusted. The results showed that the best reflection loss of −60 dB with a −10 dB range of 5.6 GHz could be obtained in an absorber with a triple-layered structure composed of GF-CNTs/epoxy with different CNT contents and GF-CNTs@Ni/epoxy. Zhao, R. et al. [33] fabricated and systematically investigated the mechanical and microwave absorption properties of MWCNTs-GF-epoxy composites. The maximum reflection loss of the composites can reach −48 dB at 24.5 GHz. The EM wave absorption of the composites strongly depends on MWCNTs and GF contents and can reach values similar to 70% at 26.5 GHz.

Usually, researchers use a combination of simulation and experimentation to comprehensively investigate the absorbing properties of absorbing composite [34,35,36]. They obtain simulation results by testing the electromagnetic parameters of the absorbents and experimental results by preparing samples, mixing the absorbent with resin and short-cut fiber [37]. However, this short-cut fiber-reinforced composite material cannot provide good mechanical strength and is rarely used in practical applications. When both mechanical strength and absorbing performance are required in materials, the utilization of continuous fibers as reinforcement materials becomes imperative. Consequently, the preparation method for absorbing composite materials is different. This involves a series of steps, including blending resin with absorbents to generate a film, followed by compositing it with continuous fiber fabric to obtain prepreg. The composite laminates are then prepared using the prepreg and cured through autoclave. Although the mass ratio of the absorber in the prepreg film can be determined at the beginning, it is essential to consider that after the curing process, part of the resin in the prepreg film flows into the fiber bundle, while the absorbent, due to its larger volume, does not penetrate inside, which will cause an increase in the mass ratio of the absorbent in the area between the fiber fabrics. The variation in the absorbent’s mass ratio inevitably induces alterations in its absorption performance. If we persist in employing the initial mass ratio of the absorbent to conduct a simulation, the outcomes will deviate from experimental results. This is an aspect that previous researchers overlooked. The primary objective of this study is to quantitatively assess and determine how much the mass ratio of absorbent increases.

In this paper, we prepared a glass-fiber-fabric-reinforced microwave-absorbing composite via a prepreg process and studied its absorption properties based on various thickness. Then, we compared the simulation results and the experiment results and determined the reason for the difference. Based on the metallographic results, we come up with three shape approximation models to calculate the mass fracture of the absorbent in the composite and obtain a better simulation result, which lays a foundation for the optimization design of the microwave-absorbing composite.

## 2. Experimental Section

### 2.1. Materials

**Figure 1:**
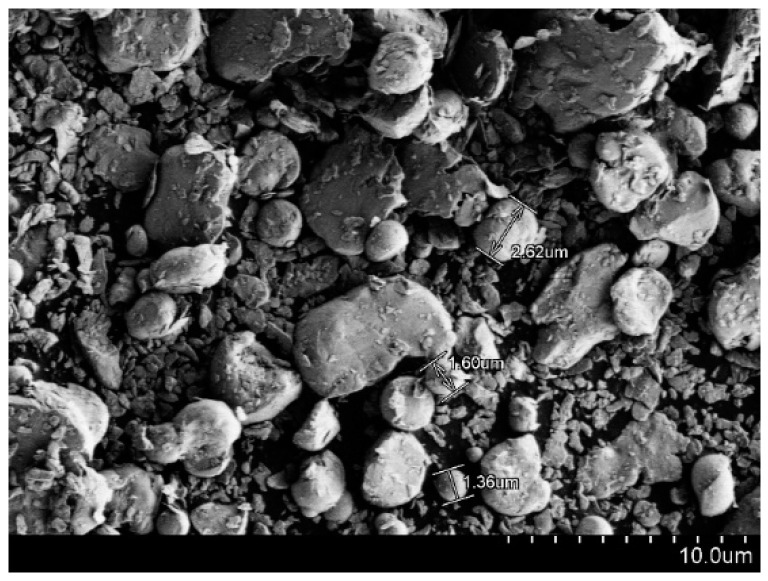
The TEM image of the absorbent.

The absorbent was supplied by Beihang University, China, and has a particle diameter of 1~7 µm, as shown in Figure 1. The material designation is BU314. The absorbent is carbonyl iron powder with both magnetic and dielectric loss mechanisms. The epoxy resin was purchased from Zhengzhou university, China, and has a density of 1.22 g/m^3^. The material designation is ME301, with a viscosity of approximately 7000 mPs at 70 °C. A 2D plain weave E-glass fabric was obtained from China Jushi Co., Ltd., Jiaxing, China, with the fabric’s density of 100 g/m^2^, and its fiber number in the latitude and longitude directions are both 20/cm. The material designation is EW100a.

Figure 1 indicates the particle size of the absorbent powder, and it is evident that this absorbent exhibits a relatively broad particle size distribution, which facilitates achieving a wide-ranging absorption performance.

### 2.2. Preparation of Absorbent/Epoxy Resin Composites

Before preparing the composites, the absorbent was dried in a vacuum oven at 60 °C for 1 h to remove moisture. The epoxy resin was then warmed to 70 °C in a beaker. After that, the absorbent powder and epoxy resin were mixed thoroughly through vigorous mechanical stirring at 70 °C for 10–15 min. The mass ratio of the absorbent powder in the composites was set at 76%.

### 2.3. Preparation of Prepreg

The resin matrix was preheated in the oven at a temperature of 60~70 °C for approximately 0.5 h and then spread onto the glass fabric to create a prepreg with a density of 320 g/m^2^ on the laminating machine at (90 ± 5 °C). This particular prepreg, labeled E1, contains absorbent and glass fabric.

### 2.4. Preparation of Composite Laminates

The prepreg was arranged in the order shown in Table 1 and then placed into a vacuum bag. The composite laminates were produced using the same curing process in an autoclave, following these steps: vacuuming the bag at room temperature to achieve a vacuum level of at least −0.095 MPa, applying pressure of 0.3 MPa at the start of the curing process, raising the temperature to 135 °C at a heating rate of 1~1.5 °C/min, maintaining this temperature for 150 min, and then lowering the temperature to 60 °C with a cooling rate of 2~3 °C/min.

Table 1 presents the ply count and lay-up configuration of each sample. The thickness of each sample is measured using a precision deep arch micrometer. The lamina thickness is determined by dividing the overall thickness by the ply count for each respective sample.

## 3. Results and Discussion

### 3.1. The Simulation Results of the Composite

According to the transmission line theory, the RL theoretical calculation equations of a single-layer absorber backed a perfect metal plate are as follows:

(1) $$RL(dB)=20{log}_{10}(\left|\frac{Z_{in}-Z_{0}}{Z_{in}+Z_{0}}\right|),$$

(2) $$Z_{in}=Z_{0}\sqrt{\mu_{r}/\varepsilon_{r}}tanh\left(\gamma d\right),$$

(3) $$\gamma =j\omega \sqrt{\mu_{r}\varepsilon_{r}},$$

where $Z_{in}$ is input impedance of the absorber, and $Z_{0}$ is the impedance of the free space about 377 Ω. The $\mu_{r}$ and $\varepsilon_{r}$ are the complex permeability and permittivity of composites. ω is the angular frequency with the unit of rad/s, and d is the thickness of the composite with the unit of mm.

**Figure 2:**
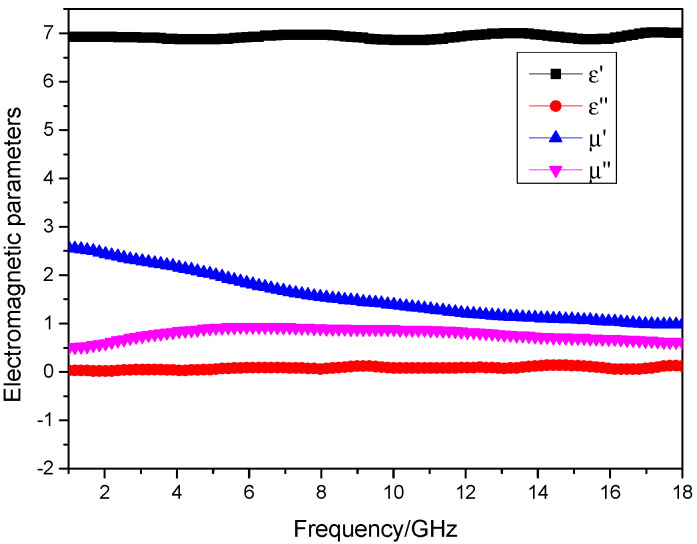
The electromagnetic parameters.

**Figure 3:**
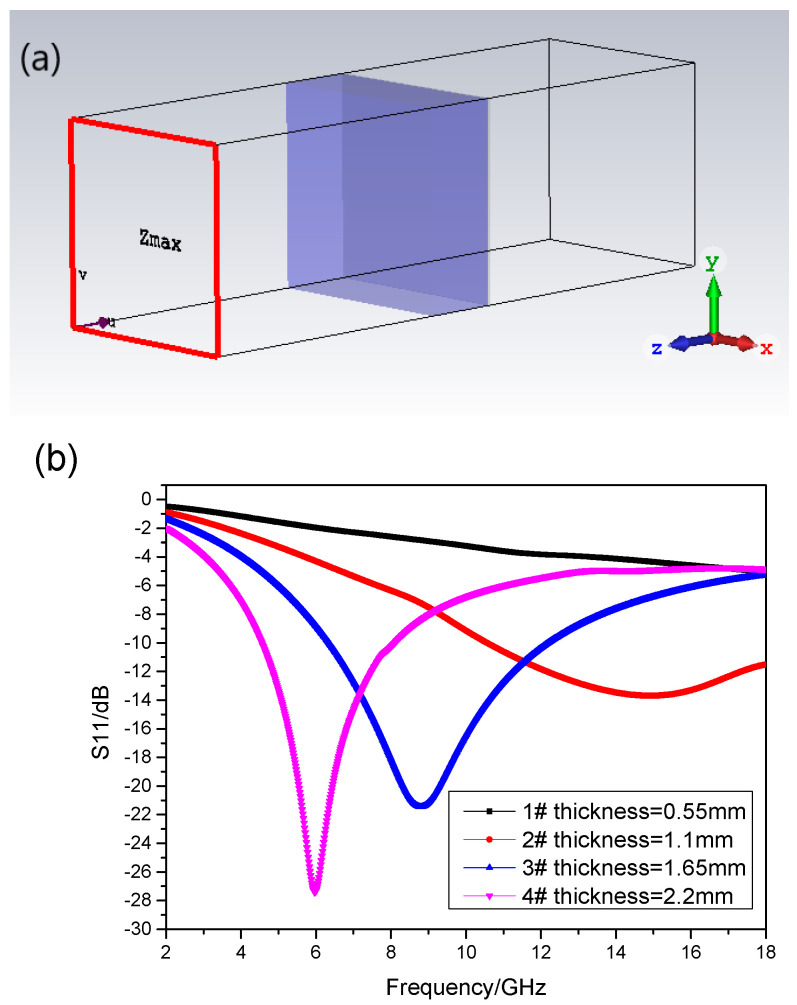
The simulation process (**a**) model (**b**) the simulation results of 1#~4# samples.

The absorbent was mixed with paraffin wax at a mass ratio of 76%, and the measured electromagnetic parameters of the material are shown in Figure 2:

Figure 2 shows that the real part of the permeability of the absorbent has the frequency dispersive characteristic, while the real part of its permittivity is approximately equal to a constant, which is 7. The imagery part of the permeability is bigger than the imagery part of the permittivity, which implies that the major absorbing mechanism is magnetic loss. Based on the calculation formula of RL (CST 2016), a commercial software is applied to do simulation. The boundary condition on the X–Y plane is set as “unit cell”, and that on Z-axis is set as “open add space”. Floquet ports are applied, and the incidence direction of the microwave is from left to right as shown in Figure 3a. Moreover, the backing of the absorber is a perfect electric conductor, which means the RL of the absorber could be presented by S11 parameter. The absorptive properties of the absorption composite with the thicknesses of 1#~4# sample can be calculated as shown in Figure 3.

Figure 3b demonstrates that the absorbing peak moves to a low frequency with the increase in the thickness of the absorbing composite. And the minimum RL also goes deeper. The minimum RL of the composite with a thickness of 1.1 mm is −13.7 dB at 14.91 GHz, and the bandwidth of RL ≤ −10 dB is from 10.56 to 18 GHz, which covers the whole Ku band. The minimum RL of the composite with a thickness of 1.65 mm is −21.44 dB at 8.8 GHz, and the bandwidth of RL ≤ −10 dB is from 6.35 to 12.1 GHz, which covers the whole X band. When the thickness increases to 2.2 mm, the minimum RL is −27.4 dB at 5.79 GHz, and the bandwidth of RL ≤ −10 dB is from 4.56 to 8.09 GHz, which almost covers the C band.

### 3.2. The Experiment Results of the Composite

**Figure 4:**
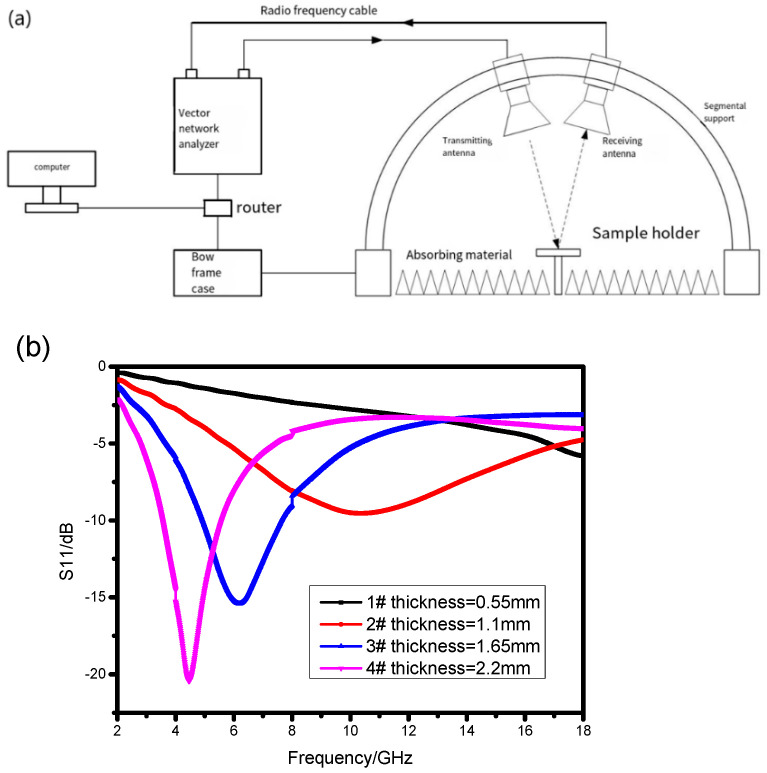
The experiment results: (**a**) the schematic diagram of the equipment; (**b**) the S11 results.

Then the arched frame equipment was applied to measure the S11 parameter of the absorber. The results are shown in Figure 4.

Table 2 illustrates the comparison of the simulation results and experimental results in terms of absorption peak position and effective absorption bandwidth. From Table 2, it can be observed that as the sample thickness increases, the difference in absorption peak position decreases. The difference in absorption peak positions for sample 2# amounts to 4.56 GHz, whereas for sample 4#, this discrepancy reduces to 1.33 GHz. Regarding the effective absorption bandwidth, sample 2# does not exhibit any effective absorption bandwidth exceeding −10 dB; however, the simulation results show that it could cover Ku band. At the same time, samples 3# and 4# also demonstrate notable deviations when compared to the simulation results.

Figure 4 displays that the experimental results share the same trend with the simulation results when the thickness of the absorbing composite increases, while the absorbing peaks and the bandwidth of RL ≤ −10 dB are different at the same parameters. The absorbing peaks in experimental results move to the lower frequency compared to the simulation results, and the absorbing band becomes narrow. We argue that the reason for the discrepancy between the experiment and the simulation is the different electromagnetic parameters, which means that the mass ratio of absorbents must increase in the experiment results. This is a reasonable result because during the manufacture process, we need to add conditions of vacuum and pressure; part of the resin in the absorbent layer on the prepreg will be extruded into the reinforced fiber bundles, resulting in a proportionate increasing of the absorbent in the absorbent layer. In order to verify our idea, we conducted a metallographic on the prepared absorbing composites.

**Figure 5:**
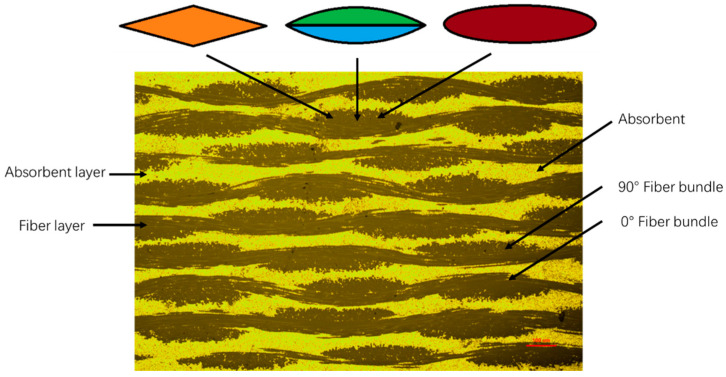
The metallographic result.

Figure 5 demonstrates the different components of the absorbing composites, in which the yellow highlight is the absorbent, the black continuum is the reinforcing fiber, and the grey dots that dispersed between fiber bundles and the absorbent is the resin. Since the fiber fabric is plain cloth, the interwoven structure of 0° and 90° fiber bundles can be observed. Meanwhile, we found that the absorbent is distributed between the fiber layers, and it cannot enter the fiber bundles. Therefore, the flow of the resin under the conditions of vacuum and pressure must increase the mass ratio of the absorbent in the absorbent layer, which leads to the change in the absorbent performance in the experiment results.

### 3.3. The Shape Approximation Methods

However, in order to optimize the simulation results, we need to quantitatively analyze the change of absorbent mass ratio after curing. From Figure 5, we can tell that the volume and mass of the absorbent layer cannot be obtained directly. Through further observation, we found that the cross-section of the 90° fiber can be seen as a certain regular shape, so the volume of a single cross-section of the 90° fiber, which contains resin after curing, can be calculated through shape approximation; the volume of the absorbent can be obtained by its mass and density; them the mass of the resin in the absorbent layer can be calculated; and the mass ratio of the absorbent layer can be finally obtained. The specific calculation process is as follows:

(4) $$A=\frac{M_{a}}{M_{r}+M_{a},}\;$$

(5) $$M_{r}=\rho_{r}\left(V_{0}-V_{f}-V_{a}\right).$$

*A* is the absorbent mass ratio in the single absorbent layer. $M_{r}$ is the mass of the resin in a single layer, $M_{a}$ is the mass of the absorbent in a single layer, $V_{0}$ is the volume of the single layer, $V_{f}$ is the volume of fiber/resin composite in a single layer, and $V_{a}$ is the volume of the absorbent in a single layer. And

(6) $$V_{a}=\frac{M_{a}}{\rho_{a}},$$

where $\rho_{a}$ is the density of the absorbent.

It can be seen from the metallographic image in Figure 5 that the shape of the cross section of a single fiber bundle can approximately be seen as parallelogram, a combination of two bows, or an ellipse.

**Figure 6:**
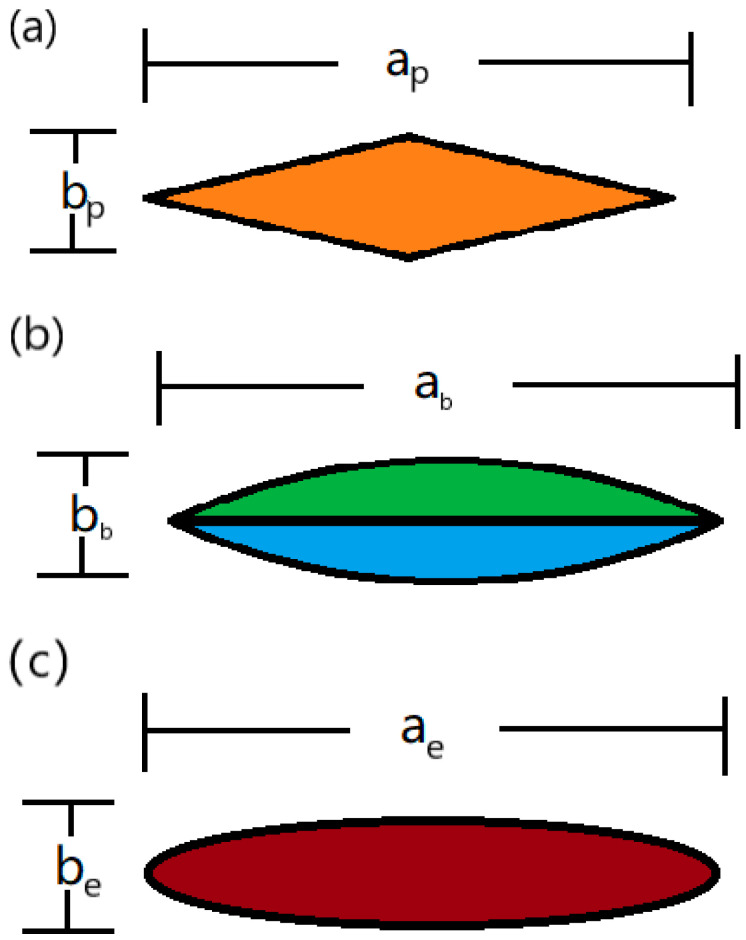
The approximation shape of (**a**) parallelogram, (**b**) a combination of two bows, and (**c**) an ellipse.

Figure 6 demonstrates three different approximation shapes of the cross section of a single fiber bundle.

If the cross section of a single fiber bundle can be approximately seen as parallelogram, as shown in Figure 6a, where the base of the triangle in the parallelogram is $b_{p}$ and the height is $a_{p}$/2, the cross-sectional area $S_{p}$ can be calculated as follows:

(7) $$S_{p}=\frac{a_{p}b_{p}}{2},$$

(8) $$V_{f}=S_{p}L_{O}n.$$

The mass ratio of the absorbent *A* can be calculated with the following formula:

(9) $$A=\frac{M_{a}}{\rho_{r}(V_{0}-\frac{a_{p}b_{p}}{2}L_{O}n-\frac{M_{a}}{\rho_{a}})+M_{a}.}$$

If the cross section of a single fiber bundle can be approximately seen as a combination of two bows, as shown in Figure 6b, where the chord length of the bow is $a_{b}$ and the bow height is $b_{b}$/2, the cross-sectional area $S_{b}$ is calculated as follows:

(10) $$S_{b}=2\left[\frac{2\theta \pi R^{2}}{2\pi }-\frac{a_{b}\left(R-\frac{b_{b}}{2}\right)}{2}\right],$$

(11) $$R^{2}-{\left(R-\frac{b_{b}}{2}\right)}^{2}={\left(\frac{a_{b}}{2}\right)}^{2},$$

(12) $$R=\frac{a_{b}^{2}+b_{b}^{2}}{4b,}$$

(13) $$\theta =arcsin(a_{b}/2),$$

(14) $$V_{f}=S_{b}L_{O}n.$$

The mass ratio of the absorbent *A* can be calculated by the following formula:

(15) $$A=\frac{M_{a}}{\rho_{r}(V_{0}-S_{b}L_{O}n-\frac{M_{a}}{\rho_{a}})+M_{a.}}$$

If it is an ellipse, where the major axis of the ellipse is a and the minor axis is b, as shown in Figure 6c, then, the formula for calculating its area is as follows:

(16) $$S_{e}=\pi \frac{a_{e}b_{e}}{4},$$

(17) $$V_{f}=S_{O}L_{O}n,$$

where n is the number of fibers and $L_{O}$ is the length of a single fiber bundle. If it is substituted into Equation (4), the mass ratio of the absorbent *A* can be calculated by the following formula:

(18) $$A=\frac{M_{a}}{\rho_{r}(V_{0}-\pi \frac{a_{e}b_{e}}{4}L_{O}n-\frac{M_{a}}{\rho_{a}})+M_{a}.}$$

Considering that the fabric fiber is not straight in the composite material, it is necessary to introduce a correction factor $K_{f}$ in the length calculation. According to the metallographic test results, the bending angle α of the fiber is about 5.089°.

**Figure 7:**
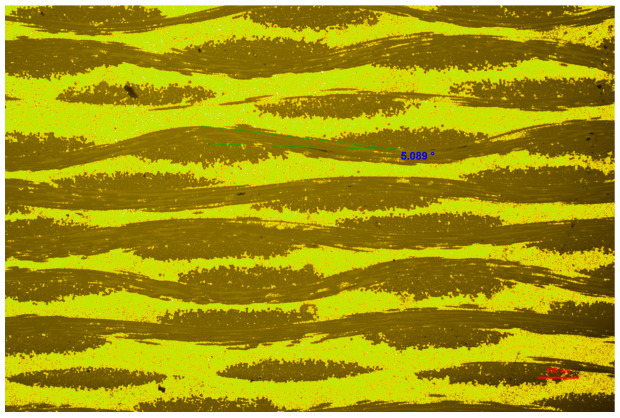
The bending angle of the fiber bundle.

Figure 7 demonstrates the bending angle of the fiber bundle, which means the fiber in the composite is longer than the size of the composite samples. Therefore, the correction factor $K_{f}$ is

(19) $$K_{f}=\frac{1}{cos\alpha }=1.003957,$$

(20) $$L_{O}={1000K}_{f}mm$$

**Figure 8:**
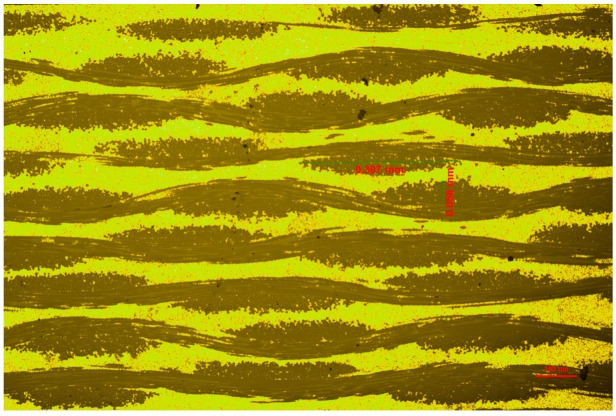
The measurement result of the structure parameters of 90° fiber bundles.

Figure 8 displays the metallographic image of the sample at the scale of 100 μm and shows the measurement result of the structure parameters of 90° fiber bundles. We substituted a = 0.397 mm and b = 0.056 mm into the above formulae to calculate the mass ratio of the absorbent. The results are shown in Table 3.

Table 3 displays the proportions of absorbers in the absorption layer obtained through different approximation methods. According to the data, the ellipse approximation method exhibits the highest proportion of absorbers, reaching 90.49%, while the parallelogram approximation method demonstrates the lowest proportion at 80.07%. Subsequently, we obtained their electromagnetic parameters based on the calculated proportions of absorbent.

**Figure 9:**
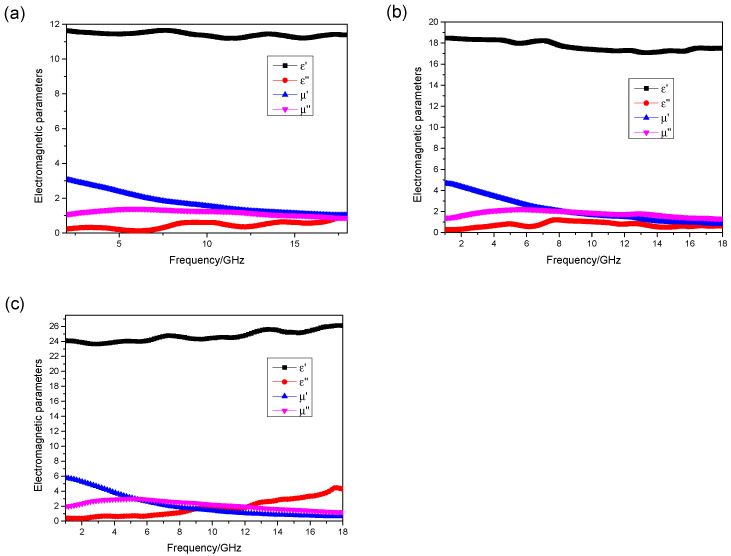
The mass ratio of the absorbent at (**a**) 80.07%, (**b**) 85.95%, and (**c**) 90.49%.

The absorbent was mixed with paraffin wax at a mass ratio of 80.07%, 85.95%, and 90.49%, respectively. The electromagnetic parameter test results are shown in Figure 9.

**Figure 10:**
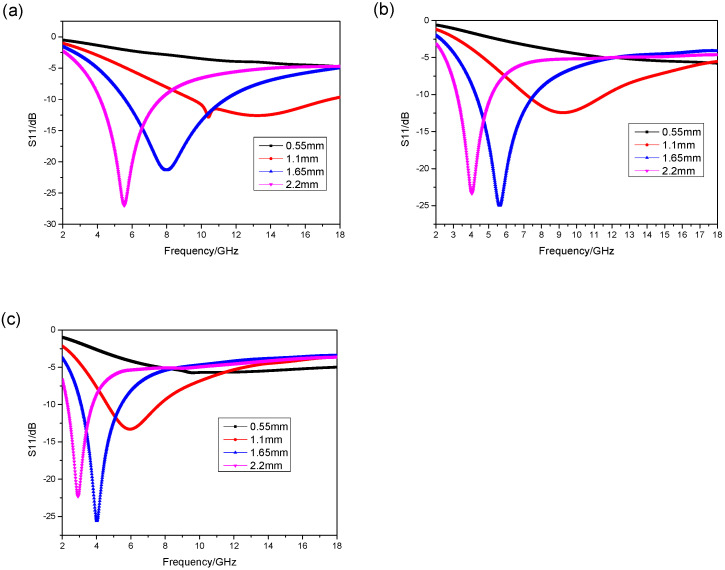
The simulation results with the mass ratio of absorbent at (**a**) 80.07%, (**b**) 85.95%, and (**c**) 90.49%.

According to the calculation formula of RL, the absorption properties can be calculated as shown in Figure 10.

Table 4 demonstrates the comparison of the simulation results and the experimental results in terms of absorption peak positions of those three different approximation methods. After each column of simulation results, the error compared to the experiment results was calculated. From the data, we can tell that the errors differentiating the simulation results of the three approximation methods from the experimental results exhibited a pattern of an initial decrease followed by a subsequent increase. Notably, the combination of two bows approximation method demonstrated the smallest error, while the ellipse approximation method yielded the largest discrepancy. After using the combination of two bows approximation method, sample 2# displayed a reduced error to 1.12 GHz; for sample 3#, it decreased to 0.56 GHz; and for sample 4#, it decreased to 0.41 GHz.

Table 5 displays the comparison of the simulation results and experimental results in terms of effective absorption bandwidth after applying the combination of two bows approximation method. According to the data, it can be observed that sample 3# has an effective absorption bandwidth of 3.29 GHz, ranging from 4.29 GHz to 7.58 GHz, which coincides with the experiment results indicating a bandwidth of 2.9 GHz from 4.93 GHz to 7.58 GHz. Similarly, for sample 4#, the effective absorption bandwidth is found to be 2 GHz, spanning from 3.2 GHz to 5.2 GHz, which also aligns with the experiment result showing a bandwidth of 2.02 GHz from 3.56 GHz to 5.58 GHz.

The comparison between Figure 3 and Figure 10 displays that the simulation results obtained via the optimated electromagnetic parameters are closer to the experimental results. Among them, the error of absorption performance calculated after ellipse approximation is the largest, and the absorping peaks all move to low frequency, which means the mass ratio of the absorbent calculated via ellipse approximation is larger than the one in reality, while the absorping peaks obtained via parallelogram approximation moves to high frequency, which means that the mass ratio of the absorbent is smaller than the one in reality. The simulation results obtained via combination of two bows approximation coincide with the experimental results, which means the mass ratio of the absorbent in the composite absorbing material raises by 9.95% after curing. By applying this change, the accuracy of simulation results is improved.

## 4. Conclusions

The glass-fiber-fabric-reinforced resin-based microwave-absorbing composite material was prepared. According to the simulation results, the bandwidth of RL ≤ −10 dB could cover Ku, X, and C bands, respectively, at different thicknesses. However, the experimental results differ from the simulation results, and the reason for the differences is the change in the mass ratio of the absorbent after the curing process. Three shape approximation methods were applied to quantitatively analyze it, and the simulation results imply that the simulation results obtained via combination of two bows approximation coincide with the experimental results, and the mass ratio of the absorbent in the composite absorbing material raises by 9.95% after curing. The results provide guidance for the subsequent design of the electrical properties of absorbing composites.

## Figures and Tables

**Table 1:** Composite laminates.

Sample	Label	Ply Count	Lay-Up	Thickness	Lamina Thickness
1#	E1	5	[0]_5_	0.55 mm	0.11 mm
2#	E1	10	[0]_10_	1.10 mm	0.11 mm
3#	E1	15	[0]_15_	1.65 mm	0.11 mm
4#	E1	20	[0]_20_	2.20 mm	0.11 mm

**Table 2:** Comparison of simulation results and experiment results. Units: GHz.

Sample	Absorbing Peak			Bandwidth of RL ≤ −10 dB	
	Simulation	Experiment	Error	Simulation	Experiment
2#	14.91	10.35	4.56	10.56~18	/
3#	8.80	6.19	2.61	6.35~12.20	4.93~7.63
4#	5.79	4.46	1.33	4.56~8.10	3.56~5.58

**Table 3:** The mass ratio of the absorbent in the absorbent layer under different approximate calculation methods.

Approximation Shape	The Mass Ratio
Parallelogram	80.07%
Combination of two bows	85.95%
Ellipse	90.49%

**Table 4:** The comparison of absorbing peaks between the different approximations simulation results and the experiment results. Units: GHz.

Sample	Experiment	Parallelogram		Combination of Two Bows		Ellipse	
		/	Error	/	Error	/	Error
2#	10.35	13.23	2.88	9.23	1.12	5.95	4.4
3#	6.19	7.98	1.79	5.63	0.56	4.03	2.16
4#	4.46	5.55	1.09	4.05	0.41	2.93	1.53

**Table 5:** The comparison of the bandwidth of RL ≤ −10 dB between the bows approximation simulation results and the experiment results. Units: GHz.

Sample	Combination of Two Bows	Experiment
2#	7.14~11.65	/
3#	4.29~7.58	4.93~7.63
4#	3.20~5.20	3.56~5.58

## Data Availability

Data are contained within the article.
